# Relative risk for cardiovascular atherosclerotic events after smoking cessation: 6–9 years excess risk in individuals with familial hypercholesterolemia

**DOI:** 10.1186/1471-2458-6-262

**Published:** 2006-10-23

**Authors:** Anneke Kramer, Angelique CM Jansen, Emily S van Aalst-Cohen, Michael WT Tanck, John JP Kastelein, Aeilko H Zwinderman

**Affiliations:** 1 Department of Medical Informatics, Academic Medical Center, University of Amsterdam, The Netherlands; 2 Department of Vascular Medicine, Academic Medical Center, University of Amsterdam, The Netherlands; 3 Department of Clinical Epidemiology and Biostatistics, Academic Medical Center, University of Amsterdam PO Box 22700, 1100 DD Amsterdam, The Netherlands

## Abstract

**Background:**

Smoking history is often di- or trichotomized into for example "never, ever or current smoking". However, smoking must be treated as a time-dependent covariate when lifetime data is available. In particular, individuals do not smoke at birth, there is usually a wide variation with respect to smoking history, and smoking cessation must also be considered.

**Methods:**

Therefore we analyzed smoking as a time-dependent risk factor for cardiovascular atherosclerotic events in a cohort of 2400 individuals with familial hypercholesterolemia who were followed from birth until 2004. Excess risk after smoking-cessation was modelled in a Cox regression model with linear and exponential decaying trends. The model with the highest likelihood value was used to estimate the decay of the excess risk of smoking.

**Results:**

Atherosclerotic events were observed in 779 patients with familial hypercholesterolemia and 1569 individuals had a smoking history. In the model with the highest likelihood value the risk reduction of smoking after cessation follows a linear pattern with time and it appears to take 6 to 9 years before the excess risk is reduced to zero. The risk of atherosclerotic events due to smoking was estimated as 2.1 (95% confidence interval 1.5; 2.9).

**Conclusion:**

It was concluded that excess risk due to smoking declined linearly after cessation in at least six to nine years.

## Background

Smoking is one of the most important risk factors for disease, particularly for lung, and cardiovascular disease (CVD) [1]. In many studies where lifetime smoking data is not available, a patient's smoking history is dichotomized into "yes or no smoking", or trichotomized into "never, ever, or current smoking". Subsequently, odds ratios or relative risks are calculated in a case-control study or cohort study, respectively. Although likely crude, the untoward effect of smoking is clearly illustrated in such analyses, provided that smoking occurred before development of disease in the case-control study, or that follow-up in the cohort study started at some point in time at which smoking history could be determined. Many prognostic models have been developed with smoking as a time-independent factor, i.e. not changing in time. Examples of such models for cardiovascular disease are the Framingham risk score [2], the Procam risk score [3], and recently the score developed by Yusuf et al. [1]. It is challenging to collect reliable data on smoking from decades ago, and this may be the reason why di- or trichotomization, is often used to summarize smoking history. But this is often suboptimal since it is likely that a person who ceased smoking ten years ago is at a different risk for developing cardiovascular disease, for instance, than a person who ceased smoking at the day follow-up started in a cohort study.

When lifetime risk data are available, the di- or trichotomization of smoking history is not appropriate, because follow-up starts at birth. This report evaluates the development of cardiovascular disease in individuals with Familial Hypercholesterolemia (FH) [4], and the use of smoking as a time-dependent variable in this study (GIRaFH study). These individuals have a mutation of the low density lipoprotein (LDL)-receptor gene which causes severely increased LDL-cholesterol levels from birth onwards, and therefore an increased risk of CVD. In this study, FH-individuals were followed from birth and all cardiovascular events were registered. The aim was to quantify the lifetime risk and the influence of classical risk factors, such as smoking, for cardiovascular disease [4]. In this study smoking could not be treated as a time-independent risk factor since individuals did not smoke at birth, and because there was very large variation with respect to the age at which smoking started, and age of smoking cessation. In the statistical analysis of such data, smoking is commonly treated as a time-dependent variable. Its value is set to zero at birth, and then changes to unity at the age at which the individual starts smoking. When the individual stops smoking, the value may be changed back to zero, which implies that the excess risk due to smoking reduces to zero immediately upon cessation. Alternatively, the value of the time-dependent variable may be kept at unity for the remaining lifetime, which implies that the excess risk of smoking remains even after cessation. The best choice is likely in between, implying that the excess risk due to smoking decreases gradually after cessation. Dobson and co-workers [5,6] performed two case-control studies in individuals with coronary artery disease, and found that the excess risk of smoking disappeared 3 to 6 years after smoking cessation. The present study analyzes the risk of cardiovascular atherosclerotic events as a function of smoking and time since smoking cessation in a cohort of 2400 individuals with FH who were followed from birth onwards.

## Methods

### Study design and study population

The GIRaFH study was a retrospective, multicenter, cohort study. The study design and study population have been described elsewhere [4]. Briefly, lipid clinics in the Netherlands submit DNA samples from clinically suspected FH patients to a central laboratory for LDL-receptor mutation analysis [7]. We randomly selected hypercholesterolemic patients from this DNA-bank database. These patients had been referred from 27 lipid clinics throughout the Netherlands. A total of 2400 FH patients were included in this study. The FH diagnostic criteria were based on internationally established criteria [8].

Phenotypic data (including detailed information on cardiovascular events) were acquired by reviewing patient's medical records by a trained team of data collectors [9]. Guidelines for data collection from medical records were constructed for the purpose of the study and have been published [9]. Written informed consent was obtained from all living patients. The Ethics Institutional Review Board of each participating hospital approved the protocol.

### Determination of smoking-history

Upon review of the patient's medical records, sufficient data on lifetime smoking status was available in the medical records of 68 percent of the patients. Questionnaires were mailed to all participants in order to supplement the smoking data. In addition to the number of cigarettes smoked per day, the questionnaire contained questions on age when the person started smoking, age(s) when smoking cessation was attempted, and age of final cessation. With the supplemental data collected from the questionnaires, lifetime smoking status could be reconstructed in 88 percent of the patients. Temporary cessations were exceptional and short, and we decided to analyze final cessation dates only. Patients of whom smoking status was unknown, had slightly higher HDL levels (1.33 vs 1.23 mmol/L, p = 0.001), and they had lower triglyceride levels (1.68 vs 1.92 mmol/L, p = 0.009). On the other covariates that we analyze in this paper the two patient groups were not significantly different.

### Other risk factors

Male gender, smoking, body mass index and the presence of hypertension, and diabetes mellitus were considered classical risk factors. Hypertension was defined when the diagnosis had been made and when anti-hypertensive medication was prescribed, or if three consecutive blood pressure measurements were > 140 mmHg systolic or > 90 mmHg diastolic. Diabetes mellitus was defined when the diagnosis had been made and medication (insulin or oral anti-diabetics) was prescribed, or by a fasting glucose of > 6.9 mmol/L. In the statistical analysis, both hypertension and diabetes mellitus were handled as binary covariates depending on age; before age of diagnosis the risk factor was considered to be absent, and after diagnosis it was considered present for the remaining life.

All laboratory parameters were measured in fasting blood samples after at least 6 weeks of withdrawal of any lipid-lowering medication. The presented values are those from as close to the first lipid clinic visit as possible, with a maximum time-span of two years. Plasma total cholesterol, high density lipoprotein (HDL) cholesterol, and triglycerides were measured by standard enzymatic methods. Low density lipoprotein (LDL) cholesterol concentrations were calculated by means of the Friedewald formula. Apolipoprotein (a) (Lp(a)) was measured with immunonephelometric methods. Plasma homocysteine was measured by high-performance liquid chromatography. These laboratory parameters were handled as time-independent covariates in the statistical analysis.

### Definition of atherosclerotic events

In this study the primary outcomes were cardiovascular mortality and non-fatal cardiovascular events. Cardiovascular events were defined as (I) acute myocardial infarction (AMI), proven by at least two of the following: (a) classical symptoms (> 15 minutes), (b) specific EKG abnormalities, (c) elevated cardiac enzymes (> 2*x *upper limit of normal); (II) percutaneous coronary intervention (PCI) or other invasive procedures; (III) coronary artery bypass grafting (CABG); (IV) angina pectoris (AP), diagnosed as classical symptoms in combination with at least one unequivocal result of one of the following; (a) exercise test, (b) nuclear scintigram, (c) dobutamine stress ultrasound, (d) a more than 70 percent stenosis on a coronary angiogram; (V) ischemic stroke, demonstrated by CT- or MRI scan; (VI) documented transient ischemic attack (TIA); (VII) peripheral arterial bypass graft (PBG); (VIII) peripheral percutaneous transluminal angioplasty (PTA) or other percutaneous invasive intervention; (IX) intermittent claudication defined as classical symptoms in combination with at least one equivocal result of one of the following: (a) ankle/arm index < 0.9 or (b) a stenosis (> 50 percent) on an angiogram or duplex scan. If information on cardiovascular events did not strictly fulfill the above-mentioned criteria, or if any suspect history, symptoms, or diagnostic evaluations were found in the medical record, the case was presented to an independent adjudication committee [9].

### Statistical analysis

We consider the risk, subsequently denoted as hazard, of first (atherosclerotic) event as a function of age: *h*(*t*) represents the hazard at age *t*. Suppose that person *i *started smoking at age *a*_0 _and ceased smoking at age *a*_1_. In this paper we will use the proportional hazards (or Cox) model and we model the hazard of an event in person *i *as follows:

• if *t *<*a*_0 _then *h*(*t*|*i*) = *h*_0_(*t*);

• if *a*_0 _≤ t <*a*_1 _then *h*(*t*|*i*) = *h*_0_(*t*) * *exp*(*β*);

• if *t *≥ *a*_1 _then *h*(*t*|*i*) = *h*_0_(*t*) * *exp*(*γ*(*t*, *a*_1_)),

where *h*_0_(*t*) is the baseline hazard-function, *β *is the log-hazard ratio due to smoking in the age-period when person *i *smoked, and *γ*(*t*, *a*_1_) is the log-hazard ratio at age *t *due to smoking-cessation at age *a*_1 _(*t *> *a*_1_). Simple choices for *γ*(*t*, *a*_1_) are (*i*) *γ*(*t*, *a*_1_) = *β*, or (*ii*) *γ*(*t*, *a*_1_) = 0. In many cases it is likely that the excess-risk due to smoking only gradually disappears, for instance as a linear function (*iii*) *γ*(*t*, *a*_1_) = *β*(1 - δ * (*t *- *a*_1_)) or as an exponential function (*iv*) *γ*(*t*, *a*_1_) = β * *exp*(-p * (*t *- *a*_1_)), where *δ *and *p *are (unknown) parameters determining the speed of decrease of excess-risk after smoking-cessation. *δ *is a slope parameter and *a*_0 _+ 1/*δ *defines age after which the excess risk of smoking equals zero. There are an infinite number of monotonic decreasing functions, but most can be approximated very well by the linear or exponential models.

Models (*i*) and (*ii*) are special cases of a proportional hazards or Cox model with a simple time-dependent covariate, and the unknown parameter *β *can be easily estimated using standard algorithms. Models (*iii*) and (*iv*) can be re-written such that they also conform to the proportional hazards model, and the unknown parameters *β*, *δ*, and *p *can be estimated using standard algorithms for the proportional hazards model. To that end the time-dependent covariate *x*_*it *_was introduced, which is equal to zero if *t *<*a*_0_, equals unity if *a*_0 _≤ *t *<*a*_1_, and equals *exp*(-p * (*t *- *a*_1_)) in the exponential model if *t *≥ *a*_1_. For the linear decreasing model *x*_*it *_equals (1 - δ * (*t *- *a*_1_)) if *t *> *a*_1 _(and *x*_*it *_≥ 0).

The hazard function can be therefore written as *h*(*t*|*i*) = *h*_0_(*t*) * *exp*(β * *x*_*it*_), which is a standard proportional hazards model with a time-dependent covariate *if p or δ is known*. Since *p *and *δ *are unknown a two-step estimation procedure was used in which *p *or *δ *was varied along a grid, and for each value of *p *or *δ *we estimated *β *using the standard algorithms (using the partial likelihood). The value of *p *or *δ *with the highest likelihood value was then finally selected. The advantage of this two-step approach was that existing software programs like SAS, SPSS, or STATA could be used. Disadvantageous of this procedure was that the unreliability of the estimates of *p *and *δ *were not automatically incorporated in the standard error of the estimate of *β*. To incorporate that uncertainty in the estimates of the standard errors we performed a 1000-fold bootstrap procedure; in each bootstrap sample we determined optimal values of *p *or *δ *as well as the associated partial likelihood estimated of *β*. Standard error of *β *was subsequently derived from the distribution of the estimates in the bootstrap samples [10].

In the Cox model we always included as covariates gender, LDL-cholesterol, HDL-cholesterol, triglyceride level, homocysteine, and Lp(a) and hypertension, and diabetes mellitus as age-dependent variables.

## Results and discussion

Cardiovascular events were observed in 779 of the 2400 individuals included in this study. Most (*n *= 661) were cardiac in origin (306 Acute Myocardial Infarction, 11 Percutaneous Coronary Intervention, 19 Coronary Artery Bypass Grafting, 325 Angina Pectoris). There were 53 patients with cerebrovascular incidents (24 stroke, 29 Transient Ischemic Attack), 69 with peripheral vascular incidents (13 Peripheral Bypass Grafting/Percutanoeus Transient Angioplasty, 56 Claudicatio Intermittens), and two patients died without prior atherosclerotic events. Age of first event varied between 18 and 85 years of age with median 46.2 years. In the individuals without events, age varied between 18 and 86 with median 45.6. Cumulative incidences at thirty, forty, fifty, sixty, and seventy years of age were 0.9%, 8.8%, 25.9%, 47.6%, and 66.5%, respectively. Univariate hazard ratios of the risk factors used in this analysis are presented in Table 1; all were statistically significant.

**Figure 1:**
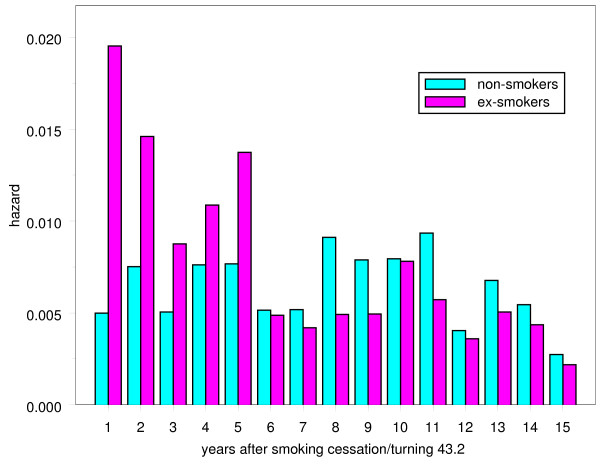
**Observed hazard for cardiovascular mortality/events in ex-smokers and in non-smokers**. Proportion of events per year since smoking cessation in persons who ceased smoking compared with a group of individuals of comparable age who never smoked.

Cardiovascular disease was increased in smokers compared to non-smokers, and this risk decreased after cessation. To illustrate this, the hazard was calculated for events in persons who ceased smoking in yearly intervals since age of smoking cessation. These hazards were compared with those in a group of individuals of comparable age who never smoked (average age of smoking cessation was 43.2 years). These hazards are given in Figure 1, and the risk is clearly increased in ex-smokers at least in the first five years after cessation. The comparison is, however, uncontrolled for other risk factors.

**Figure 2:**
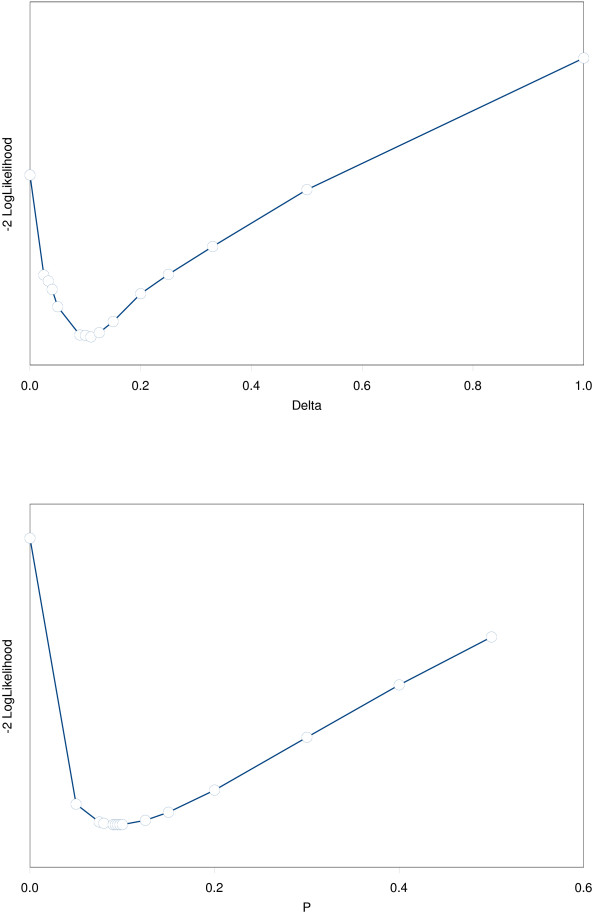
Deviance (-2 * Partial loglikelihood) values as a function of the parameters *δ *or *p *in the models with linearly or exponentially decreasing risk after smoking-cessation. Deviance values for the models with linear and exponential decreasing hazard after smoking-cessation as a function of the regression parameters in both models (*δ *and *p*).

**Figure 3:**
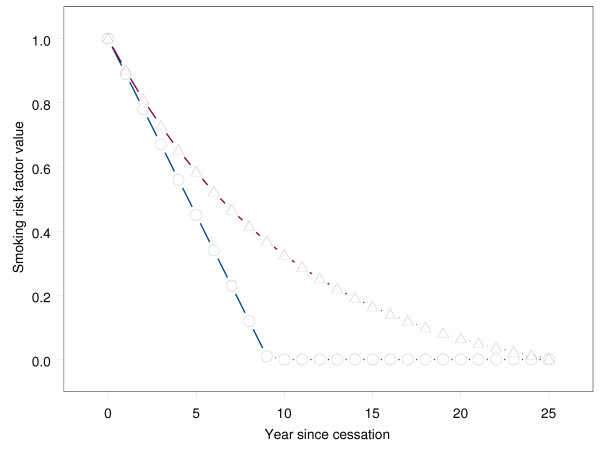
**Excess risk after smoking-cessation according to linearly and exponentially decreasing models (for the estimated parameters **δ^
 MathType@MTEF@5@5@+=feaafiart1ev1aaatCvAUfKttLearuWrP9MDH5MBPbIqV92AaeXatLxBI9gBaebbnrfifHhDYfgasaacH8akY=wiFfYdH8Gipec8Eeeu0xXdbba9frFj0=OqFfea0dXdd9vqai=hGuQ8kuc9pgc9s8qqaq=dirpe0xb9q8qiLsFr0=vr0=vr0dc8meaabaqaciaacaGaaeqabaqabeGadaaakeaaiiGacuWF0oazgaqcaaaa@2E68@**= 0.11 and **p^
 MathType@MTEF@5@5@+=feaafiart1ev1aaatCvAUfKttLearuWrP9MDH5MBPbIqV92AaeXatLxBI9gBaebbnrfifHhDYfgasaacH8akY=wiFfYdH8Gipec8Eeeu0xXdbba9frFj0=OqFfea0dXdd9vqai=hGuQ8kuc9pgc9s8qqaq=dirpe0xb9q8qiLsFr0=vr0=vr0dc8meaabaqaciaacaGaaeqabaqabeGadaaakeaacuWGWbaCgaqcaaaa@2E25@**= 0.095)**. The excess-risk after smoking-cessation according to the linear and exponential deceasing models.

The estimated rates of decline per year in excess risk after smoking-cessation according to a linear model (iii: parameter *δ*), or to an exponential model (iv: parameter *p*) are given in Table 2, together with the log hazard ratio af events due to smoking (*β*). Lowest deviance values (-2*loglikelihood) of the linear and exponential models were found for δ^
 MathType@MTEF@5@5@+=feaafiart1ev1aaatCvAUfKttLearuWrP9MDH5MBPbIqV92AaeXatLxBI9gBaebbnrfifHhDYfgasaacH8akY=wiFfYdH8Gipec8Eeeu0xXdbba9frFj0=OqFfea0dXdd9vqai=hGuQ8kuc9pgc9s8qqaq=dirpe0xb9q8qiLsFr0=vr0=vr0dc8meaabaqaciaacaGaaeqabaqabeGadaaakeaaiiGacuWF0oazgaqcaaaa@2E68@ = 0.11 and p^
 MathType@MTEF@5@5@+=feaafiart1ev1aaatCvAUfKttLearuWrP9MDH5MBPbIqV92AaeXatLxBI9gBaebbnrfifHhDYfgasaacH8akY=wiFfYdH8Gipec8Eeeu0xXdbba9frFj0=OqFfea0dXdd9vqai=hGuQ8kuc9pgc9s8qqaq=dirpe0xb9q8qiLsFr0=vr0=vr0dc8meaabaqaciaacaGaaeqabaqabeGadaaakeaacuWGWbaCgaqcaaaa@2E25@ = 0.095. Both models had much lower deviance than the models (i) with constant remaining excess risk or (ii) with no excess risk after cessation. The linear model at the optimal value of the slope parameter (δ^
 MathType@MTEF@5@5@+=feaafiart1ev1aaatCvAUfKttLearuWrP9MDH5MBPbIqV92AaeXatLxBI9gBaebbnrfifHhDYfgasaacH8akY=wiFfYdH8Gipec8Eeeu0xXdbba9frFj0=OqFfea0dXdd9vqai=hGuQ8kuc9pgc9s8qqaq=dirpe0xb9q8qiLsFr0=vr0=vr0dc8meaabaqaciaacaGaaeqabaqabeGadaaakeaaiiGacuWF0oazgaqcaaaa@2E68@ = 0.11) had slightly lower deviance than the exponential model, but the difference was small. Deviance values differed only slightly along a large range of values of *δ *or *p*, indicating that these parameters were difficult to identify despite the large number of patients, the large number of events, and the large number of exposed individuals: see Figures 2a and 2b. The excess-risk five or more years after cessation was especially uncertain, because the linear and exponential decreasing models had a similar pattern up to about five years after cessation, but diverged afterwards (see Figure 3). Some idea about the uncertainty of δ^
 MathType@MTEF@5@5@+=feaafiart1ev1aaatCvAUfKttLearuWrP9MDH5MBPbIqV92AaeXatLxBI9gBaebbnrfifHhDYfgasaacH8akY=wiFfYdH8Gipec8Eeeu0xXdbba9frFj0=OqFfea0dXdd9vqai=hGuQ8kuc9pgc9s8qqaq=dirpe0xb9q8qiLsFr0=vr0=vr0dc8meaabaqaciaacaGaaeqabaqabeGadaaakeaaiiGacuWF0oazgaqcaaaa@2E68@ or p^
 MathType@MTEF@5@5@+=feaafiart1ev1aaatCvAUfKttLearuWrP9MDH5MBPbIqV92AaeXatLxBI9gBaebbnrfifHhDYfgasaacH8akY=wiFfYdH8Gipec8Eeeu0xXdbba9frFj0=OqFfea0dXdd9vqai=hGuQ8kuc9pgc9s8qqaq=dirpe0xb9q8qiLsFr0=vr0=vr0dc8meaabaqaciaacaGaaeqabaqabeGadaaakeaacuWGWbaCgaqcaaaa@2E25@ can be obtained from Figure 2, but this uncertainty was not incorporated in the estimators of the standard errors of β^
 MathType@MTEF@5@5@+=feaafiart1ev1aaatCvAUfKttLearuWrP9MDH5MBPbIqV92AaeXatLxBI9gBaebbnrfifHhDYfgasaacH8akY=wiFfYdH8Gipec8Eeeu0xXdbba9frFj0=OqFfea0dXdd9vqai=hGuQ8kuc9pgc9s8qqaq=dirpe0xb9q8qiLsFr0=vr0=vr0dc8meaabaqaciaacaGaaeqabaqabeGadaaakeaaiiGacuWFYoGygaqcaaaa@2E64@ in Table 2. To evaluate this effect 1000 bootstrap samples were drawn from the cohort of 2400 patients, and *δ*, and *β *were estimated in each bootstrap sample. The bootstrap standard deviation was only slightly larger than the maximum partial loglikelihood estimate of the standard error in Table 2: 0.079 versus 0.077.

On the basis of this estimate of δ^
 MathType@MTEF@5@5@+=feaafiart1ev1aaatCvAUfKttLearuWrP9MDH5MBPbIqV92AaeXatLxBI9gBaebbnrfifHhDYfgasaacH8akY=wiFfYdH8Gipec8Eeeu0xXdbba9frFj0=OqFfea0dXdd9vqai=hGuQ8kuc9pgc9s8qqaq=dirpe0xb9q8qiLsFr0=vr0=vr0dc8meaabaqaciaacaGaaeqabaqabeGadaaakeaaiiGacuWF0oazgaqcaaaa@2E68@ and its standard error, the hazard ratio (HR) of smoking was estimated as 2.01 with 95% confidence interval (CI) 1.72; 2.34. After controlling for the risk-factors in Table 1 the smoking hazard ratio was 2.02 (95% CI: 1.44; 2.84). The optimal value for *δ *was estimated in the multivariable model as δ^
 MathType@MTEF@5@5@+=feaafiart1ev1aaatCvAUfKttLearuWrP9MDH5MBPbIqV92AaeXatLxBI9gBaebbnrfifHhDYfgasaacH8akY=wiFfYdH8Gipec8Eeeu0xXdbba9frFj0=OqFfea0dXdd9vqai=hGuQ8kuc9pgc9s8qqaq=dirpe0xb9q8qiLsFr0=vr0=vr0dc8meaabaqaciaacaGaaeqabaqabeGadaaakeaaiiGacuWF0oazgaqcaaaa@2E68@ = 0.18, and the estimated smoking hazard ratio at that *δ*-value was slightly larger: 2.06 (95% CI: 1.47; 2.87).

## Conclusion

Smoking cessation in individuals with Familial Hypercholesterolemia was associated with considerable decrease of the risk of cardiovascular atherosclerotic events. The risk reduction proved to follow a linear pattern over time, but it was difficult to distinguish this from nonlinear patterns. The slope of the linear reduction-line was estimated as 0.11 univariately, and 0.18 when corrected for confounders. This means that it took 1/0.18 ≈ 6 to 1/0.11 ≈ 9 years after cessation before the risk was reduced to the level of persons who never smoked.

The risk of atherosclerotic events due to smoking was estimated at 2.1 (95% ci: 1.5; 2.9), which coincides with many results in the literature [11-13], or only slightly higher [14-16]. We found only weak relationships between atherosclerotic events and number of cigarettes smoked, but this is probably due to the unreliability of these data (20 percent of the patients were not willing or able to quantify the number cigarettes smoked).

The estimate that it takes six to nine years after smoking cessation before the risk of atherosclerotic events is reduced to the level of persons who never smoked, agrees with findings by others. McElduff et al [6] found that the excess risk for a major coronary event due to smoking disappeared about four to six years after smoking-cessation in a population-based case-control study of 5,572 cases and 6,268 controls. Wannamethee et al [11] observed a similar stroke-risk reduction in 7,735 middle aged men, although the speed of reduction depended on smoking-quantity. In a population-based cohort of 758 women, Witteman et al [12] found that the excess risk for calcific deposits in the abdominal aorta was increased up to 10 years after cessation. Negri et al [13] also observed that it took up to 10 years after cessation before risk of acute myocardial infarction was reduced to the level of never-smokers in 916 cases with acute myocardial infarction and 1106 controls. A slightly longer period was observed by Kawachi et al [17] in the Nurses' Health Study among 117,001 nurses; they found that the risk of total coronary heart disease reduced by one third within 2 years of cessation, but afterwards it took 10 to 14 years for this excess risk to return to the level of those who never smoked. Chaturvedi et al [18] also observed a longer period (> 10 years) for the excess mortality-risk returned to normal in 4,427 individuals with diabetes. Omenn et al [19] observed that this excess risk was increased up to 20 years after cessation in 21,112 men and women evaluated with coronary angiography. A far shorter period of three years was found, however, by Rea et al [14] in a cohort of 2,169 persons who survived after first myocardial infarction. The variation in speed of risk-reduction after cessation between all these studies is probably caused by the differences in populations. In our population of familial hypercholesterolemia atherosclerosis is known to develop earlier in live with an increased speed of progression. It is possible that exposure to smoking has a bigger impact, and therefor takes longer to diminish. But next to differences in populations between the above mentioned studies, the variation is, in our view, also illustration of the difficulty to estimate the pattern with which excess risk reduces after cessation. Moreover, the lifetime smoking history was reconstructed from retrospective review of medical records and patient recall -both are known to be of poor quality-, and this will also influence outcome of our study.

We used the Cox regression model with smoking as a time-dependent covariate for statistical analysis of the risk after smoking-cessation. The time dependence of the excess-risk after smoking-cessation was modelled with a linear or exponential decaying trend; these two models were very flexible and capable of many possible patterns, but even more flexibility may be obtained with nonparametric techniques such as splines, kernels functions or lowess regression. We doubt whether such techniques offer much more insight because we found that it was already very difficult to distinguish different linear and (parametric) nonlinear patterns; even more irregular patterns will be even harder to distinguish. Others [14,15] used data-driven methods to estimate the excess-risk in ex-smokers in one, two, three, and up to ten years after cessation. Such statistical modelling is nonparametric in a way, since no assumption is made on the way the risk reduces after smoking-cessation, but this approach requires more parameters to be estimated, and therefore introduces more uncertainty.

We used a bootstrap approach to account for the uncertainty regarding the smoking-risk reduction after cessation. This offers the possibility to use standard statistical packages and tools for analyses. In our data the uncertainty on the smoking-risk-reduction after cessation had little effect on the estimates of the log-hazard ratio's of the Cox model of smoking itself, and of other covariates in the model, but this is probably due to the relatively large sample size and event number in our sample. Finally, we checked the proportional hazards assumption of the Cox regression model with regard to smoking by extending the model with the interaction of smoking and log(time), and found little evidence for violation of this assumption (*p *= 0.88). This is somewhat surprising since the relative risk of atherosclerotic events due to smoking is strongly related to age ([20]), but this may be due to the somewhat smaller age-distribution in our study.

In conclusion, smoking should be treated as a time-dependent risk factor when using lifetime data. Moreover, the risk reduction of smoking after cessation should be taken into account.

## Competing interests

The author(s) declare that they have no competing interests. The study was funded by the Academic Medical Center of the University of Amsterdam, the Netherlands

## Authors' contributions

AK, MWT and AHZ performed the statistical analysis, ACMJ and ESvA-C designed the study, and sampled all data, and were responsible for the clinical interpretation of all results, and JJPK supervised the entire project. All authors contributed drafting the manuscript.

## Pre-publication history

The pre-publication history for this paper can be accessed here:



## Figures and Tables

**Table 1:** Demographic and biochemical characteristics of the patients in the database and univariate hazard ratio for first atherosclerotic event

Risk factor	value	Univariate HR (95% CI)
Gender (% men)	49.2	2.95 (2.54; 3.43)
Hypertension^1 ^(%)	10.0	1.44 (1.17; 1.78)
Diabetes Mellitus^1 ^(%)	5.8	2.38 (1.49; 3.83)
LDL-C (mmol/L): mean (SD)	6.58 (1.86)	0.95 (0.91; 0.99)
HDL-C (mmol/L): mean (SD)	1.23 (0.35)	0.32 (0.26; 0.41)
Triglycerides (mmol/L): mean (SD)	1.70 (1.02)	1.14 (1.07; 1.22)
Homocysteine (*μ*mol/L): mean (SD)	12.4 (8.9)	1.08 (1.04; 1.11)^2^
Lp(a) (mg/L): mean (SD)	338 (419)	1.04 (1.02; 1.06)^3^

^1 ^Hypertension and Diabetes Mellitus were analyzed as time-dependent covariates. ^2 ^Hazard-ratio per 5 *μ*mol/L increase Homocysteine. ^3 ^Hazard-ratio per 100 mg/L increase Lp(a). HDL-c = high density lipoprotein cholesterol; LDL-C = low density lipoprotein cholesterol; Lp(a) = apolipoprotein(a); HR = hazard ratio.

**Table 2:** Deviance, and smoking log-hazard ratio (HR) plus standard error (SE) in the different models

model			deviance	log(HR) (SE)
(i)	constant excess risk		10455.93	0.643 (0.083)
(ii)	no excess risk		10542.84	0.188 (0.075)
(iii)	linearly decreasing risk	*δ *= 0	10455.93	0.643 (0.083)
		*δ *= 0.05	10441.41	0.718 (0.082)
		*δ *= 0.09	10438.31	0.708 (0.079)
		*δ *= 0.10	10438.26	0.705 (0.079)
		*δ *= 0.11	10438.09	0.704 (0.077)
		*δ *= 0.15	10438.56	0.689 (0.077)
		*δ *= 0.20	10439.79	0.672 (0.077)
		*δ *= 0.25	10444.98	0.660 (0.076)
(iv)	exponentially decreasing risk	*p *= 0	10455.93	0.643 (0.083)
		*p *= 0.05	10439.82	0.758 (0.087)
		*p *= 0.09	10438.57	0.743 (0.084)
		*p *= 0.095	10438.56	0.741 (0.083)
		*p *= 0.10	10438.58	0.738 (0.083)
		*p *= 0.125	10438.84	0.728 (0.082)
		*p *= 0.15	10439.33	0.719 (0.081)
		*p *= 0.3	10443.87	0.678 (0.078)

All p-values < 0.001, except for model (ii) *p *= 0.012. Standard errors were calculated using *δ *and *p *as given constants.
